# Translations from Foreign Exchanges

**Published:** 1882-11

**Authors:** 


### Translations from Foreign Exchanges.

---

RUPTURE OF THE UTERUS AND ITS PREVENTION IN LABOR. By DR. HOUSMANN, of Berlin, Germany.

The attention which lately has been directed to the stretching of the cervical canal and the lower part of the uterus as the principal cause of rupture of the uterus, has enriched our knowledge of the mechanism of this proceeding, but it has not entirely given the explanation of all causes in these accidents. For instance, Hofmeier could not detect any lesions of this part in the autopsy of a woman who had died from rupture of the uterus, and who had borne four children. The stretching of the canal often changing, according to the casualties in the act of labor, it requires different means to prevent the rupture of the uterus. I have observed instances of imminent rupture in two confinements of the same woman, and I have prevented the rupture in both cases by a suitable change of position. Mrs. X., a normally built and well developed woman, has had neither rachitis nor any other disease of the pelvic bones, but she has suffered from an inflammation of the genital tract five years before marriage, from which remained a chronic endometritis. Married when twenty-three years of age, she became pregnant after about nine months. On the sixth day of January, I found the head of the child very movable, a circumstance rather uncommon in a primipara. Two days after labor, pains commenced, and at noon of the same day, I found third position of the head, os-externum dilated to two cm. in diameter. Pulsations of the fœtal heart, 138 on the right side. The pains being slow, the woman walked around. But at night the contractions became severe, and in the meantime the abdomen was very sensitive. I found the head progressed into the vagina, but covered with a delicate membrane. The os-externum was situated behind the fœtal head, and high above the narrow posterior part of the vagina. It remained flabby even in a pain, except the anterior lip, which was pulled down by the descending head, the latter being covered, as usual, by the membranes. According to the examination, there was no doubt that the anterior part of the cervix uteri, and also the anterior vaginal wall were stretched downwards to a considerable degree, the head being felt through these membranes; there was imminent danger of rupture of the anterior uterine wall. The pelvis was normal, the measures being as follows: Spr. f., 25.5 cm., inbr. f. 29.0 cm., trochant; 31.5 cm., diameter 20.5 cm. There was no hydrocephalus of the child's head, and the fœtus could be felt through the abdominal walls as being without any deformities. There was no spasm of the external or internal orifice. I ordered the woman to take a sitting position, in order to retract the head into the uterus, but there was not the slightest change, the labor pains becoming severer, the anterior wall of the uterus and vagina being thinner and more painful. I now ordered the knee-elbow position, with deeply retracted shoulders, for the purpose of thus bringing the fundus uteri and the buttocks of the fœtus in front, and the head in the axis of the pelvis, the stretched parts being thus released from the pressure.

The success of this procedure was complete, and the stretching relaxed; the pains were normal, the os-externum dilated, and after three hours the woman could take the regular position. The next morning the fœtal membranes ruptured, the fœtal head descended but slowly in the œdematous infiltrated vagina, and when the heart sounds became less than 100, I extracted the asphyxiated child with the forceps. The circumference of the brachycephalic head was 33.5 cm., and the boy has yet, to-day, the remains of ecchymosis on his right foot, as a result of extravasation of blood in beginning asphyxia. A slight rupture of the perineum was healed by two sutures, *prima intentione*. A following perimetritis on the anterior uterine wall was treated with leeches, ice and injections of a 2 per cent. carbolic solution. Six weeks after confinement the menses reappeared, and six months afterward the woman became pregnant again, but she had an abortion at the end of the second month, while traveling in the mountains. In October, 1880, the menstruation ceased again, and in July of the next year labor pains set in. The examination showed the same phenomena as the first time, and I immediately ordered the knee-elbow position. Then the labor pains set in rapidly, and a healthy girl was born spontaneously. The woman had no trouble except a slight catarrh of the bowels, and later, a mastitis. The case is similar to those of Bandl, Hofmeier and Trommel, being a stretching of the cervix and the lower part of the uterus. But here there was a young woman with a normal pelvis, a normally built child, no spasm of the orificium and normal labor pains. The very extensive stretching of the anterior vaginal wall caused œdema of the same, which, in return, acted as an impediment in the regular course of the labor, preventing the turning of the fœtal head forward, and forcing me to take refuge to the forceps. The case shows also that this accident will reappear in a succeeding confinement, and it is worth remarking that both times the fœtal head was directed into the axis of the pelvis by suitable change of position, thus disburdening the extensively stretched anterior uterine and vaginal walls The cause of the deviation of the child's head is found, I think, in the inflammatory processes before marriage, which caused a fixation and shortening of the posterior cervical wall, preventing complete dilatation. But it is strange that this accident does not occur oftener, the inflammatory processes existing, doubtless, in a large number. On the other hand, I think it possible that a retarded development of the anterior cervical wall existed, directing the fœtal head to this part. After all the experiences in these cases, I think it probable that a succeeding confinement will be accompanied by the same accident, in which case the position of the parturient woman has to be changed, as stated above.

TERTIARY SYPHILIS.

The symptoms of tertiary syphilis change according to the seat of the lesions. The mouth, the nose and the nasal cavities are often the seat of the most detrimental lesions. There is either a diffuse suppuration, which destroys entirely the affected parts, or there are small tubercles. The osseous tissue can be affected in the same way as in ozœna. The ulcerations produce a purulent secretion striated with blood, and of a very disagreeable odor. Later a true necrosis of the bones sets in, which fall out, or, as in other cases, they remain, but the suppuration is continued. The effects are terrible. There is perforation of the nasal cavity and necrosis of the nasal bones, which gives the patient the characteristic appearance. Upon perforation of the palate, the mouth communicates with the nasal cavities, which gives the patient the peculiar nasal voice, and the food enters into the nasal cavities. In the posterior part of the pharynx, the ulcerations cause obstruction of the posterior nasal cavities. The œsophagus is not often the seat of syphilitic lesions, but there is sometimes cicatricial induration and contraction. The diagnosis is difficult in these cases, and it has to depend upon other phenomena. Syphilis of the stomach, pancreas or intestines is rather rare. Syphilis of the rectum is more common in females than in males, which is due to the neighborhood of the vagina and the frequent nymphomania in these cases. These tertiary accidents produce a narrowing of the intestines. If iodide of potassium acts successfully in these cases, then the diagnosis of syphilis is correct. Among the glands connected with the digestive system, the liver plays a great role. The coats of the liver are mostly attacked; there is enlargement of the constituent parts of the organ. Hyperæmia and cellular proliferation are followed by fibrous transformation. The phenomena are similar to that of cirrhosis. There is atrophy of the active elements, Glisson's capsule is engorged and there are adhesions. The neoplasma is diffuse; there are small tumors, and the hepatic cells undergo fatty degeneration. The icterus is not always due to syphilis, it coincides with the enlargement of the liver and with the troubles of the stomach and the intestines. Icterus is always dangerous in tertiary syphilis, a cirrhotic state of the liver with albuminuria being then present. There is also a fibrous retraction of the whole organ. There are found gummata and nodosities. The larynx is often attacked by tertiary syphilis, but the lesions are superficial, the voice being hoarse; there can be entire absence of the voice without pains In tertiary syphilis there are gummatous tubercles, suffusion of the glottis, and of the vocal cords. The new growth causes narrowing of the larynx; an active hyperemia produces congestion in these parts; there is œdema and impeded circulation to such a degree that the patient may become asphyxiated. In these cases, tracheotomy should be performed. These lesions are all very dangerous, and the treatment should be energetic. The affections are very complex; ulceration, necrosis, destruction of the cartilages, laryngeal and perilaryngeal phlegmon may occur. The nervous centers are equally affected. The meninges, the dura mater, the pia mater, and arachnoidal membrane are the seat of the lesions, as well as the brain substance. The meninges are inflamed and often adherent; they form a yellowish swelling which adheres to the cortical substance, and are diffused over the brain substance. The gummatous tumors are not so frequent around the cerebral mass, they are seen more often on the base of the chiasma of the optic nerve. There is a yellowish mass in the center, and a fatty one on the periphery. The true lesion of the cerebellum is produced by round tumors, and the morbid progress has three phases, that of congestion, hyperæmia and destruction. But before the diseased part is entirely destroyed, it passes over into a state of softening. The syphilis affects the arteries of the brain; the caliber of the vessel is narrowed, as in atheroma. There is impeded nutrition of the brain; the afflux of blood is therefore diminished, and sometimes there is thrombosis of the basis cranii. If there are nervous troubles, not ordinary in common cases, it will be necessary to look for other syphilitic symptoms. If there is hemiplegia suddenly on the right side, the patient being conscious, and if sensibility is not so much disturbed as in common hemiplegia, if there is diplopia, deviation of the eyes, dilatation of the pupils and incomplete aphonia; when the patient speaks imperfectly; when he cannot compose words, nor express himself except by mimics or by writing, it is safe to direct the treatment against tertiary syphilis. Headache, with aggravated pain, sometimes taking the form of delirium, and this mostly at night, is another symptom of cerebral affection in this disease. The genital organs are in a state of weakness. The troubles of vision, diplopia, dilatation of the pupils, vertigo, and progressive loss of memory are the phenomena of tertiary syphilis. Epilepsy due to syphilis is accompanied by paralysis and deviation of the eye. Syphilitic paralysis is merely a pseudo-paralysis; the weakness of the mental faculties is less than in general paralysis, and there is no delirium. The treatment consists of large doses of iodide of potassium and frictions with mercury ointment.—*Le Praticien.*

### PERFORATION OF THE ŒSOPHAGUS BY A SWALLOWED PIECE OF GLASS—EXTRACTION OF THE SAME THROUGH THE ANTERIOR WALL OF THE ABDOMEN; RECOVERY.

The patient, a merchant, twenty-seven years of age, swallowed a piece of glass by hastily drinking a glass of beer. There immediately followed a severe pain in the upper part of the œsophagus, and an hour afterwards a severe cough. Afterward, all the symptoms of stenosis appeared, solid food could not be swallowed, and often not even liquids. One year after the occurrence a high fever set in, and a cupful of pus was expectorated. The formerly healthy and muscular man became emaciated. Two years after the accident, he entered the Augusta Hospital, at Berlin. The most striking phenomenon was a severe and continuous cough on pressure at the epigastrium, and there was a very sensitive spot on the ensiform appendix of the sternum. On first admission liquids were swallowed easily, but after two weeks everything was vomited, and the patient had to be fed by the œsophageal tube. In introducing this tube, there were severe pains when the tube reached the cardia. The food was easily digested. Thus the patient was kept for two months, and the cough was relieved by hypodérmic injection of five drops of chloroform into the epigastric region, but gangrene of the skin prevented the continuation of the injections. When the slough of this gangrenous spot was removed, there the long missed piece of glass was seen, looking through the abdominal wall, and it was at once removed by a small forceps. It consisted of a triangular piece of window glass, with sharp pointed edges, 1½ cm. long and ½ cm. on the basis. The cough disappeared entirely, but it took about three months before the patient could take and retain solid food. After this time he recovered entirely.—*Med. Chir. Centrblatt.*

PERMANENT DRAINAGE IN HYDATIDS OF ECHINOCOCCUS.

A young farmer woman was affected with a large abdominal tumor, and by aspiration a liquid was drawn off, filled with hydatids. After puncture the cyst became smaller by spontaneous insulation. On the thirteenth day, severe pains set in, and the tumor became as large as before. Now a trocar of medium caliber was introduced, and 5 liters of a turbid liquid withdrawn. A rubber tube, with a bag attached, was then inserted, the end of which was conducted into a basin filled with water. A suitable bandage was applied to make the tube immovable ; thus a continuous aspiration was effected. About 66 ounces were withdrawn afterwards, and in twenty days the patient left the hospital entirely cured. This procedure shows that an echinococcus cyst may be cured without incision, without application of caustics, and without causing the walls to adhere to the neighboring organs. Aspiration thus performed causes cicatrization of the cyst, it impedes the entrance of air, and it is without any danger.—*Gazetta Medica Italiana.*

VERTIGO DE MENIÈRE.

The patients afflicted with this disease are able to tell their own story, because they do not become completely unconscious. In the moment of the attack they hear a noise like that of an engine, and then they fall down forward, as if struck by a superior force, the latter being often so strong as to cause bruises of the nose or loss of teeth. After a while they will rise again and begin to vomit ; they fall into a stupor, which lessens by degrees. After one or two weeks the attack will be repeated with the same phenomena. In a certain number of cases, the disease appears in the above described manner ; the patients being well the rest of the time. But in a good many cases a permanent vertigo exists, with constant noises in the ear like that of a drum or a whistle. When the noise is aggravated, the attack will follow

M. Charcot has at the present time a patient who has been in bed for the last five years, and who avoids the least movement, which latter produces a feeling as if she would be raised in the bed very quick and then be lowered just as quickly. The noise may be due to an accumulation of cerumen in the ear, in which case the removal removes the noise. But more often it is due to simple otitis, or to another affection in the inner ear. M. Charcot treats these cases by the use of quinine, and says that this is a sure remedy.—*Journ. de Médic. et Chirurg. Prat.*

### ON OSTEOTOMY AND TARSOTOMY IN CLUB-FOOT.

The surgeons of all countries, and of good authority, practice in cases of club-foot, ablation of the astragalus, scaphoideus or cuboideus; others perform resection of the same bones, or even of the calcaneus, and that for all the different kinds of club-foot in children, as well as in adults. This practice M. Jules Guérin thinks useless and detrimental to the affected persons. The surgeons who perform tarsotomy ignore the fact that the veritable club-foot is due to muscular retraction in the limb and foot. The only means to reduce all forms of club-foot, whatever its mechanical etiology may be, are tenotomy, manipulations and surgical apparatus. By tenotomy, M. Jules Guérnin, in 157 cases, has completely cured 61 ; improved, 49, the rest having not remained under treatment, or died from accidents. Old cases of club-foot which have remained from the time before orthopædic surgery was introduced, it is better to let alone, these persons being used to progression in their own way. Tarsotomy offers another impediment in the treatment of club-foot, the articular surfaces and the normal connections having disappeared. There is a certain class of deformities resulting from osteitis, osteoarthritis, strumous deformations and necrosis where tarsotomy can be applied, but these do not belong to club-foot.—*Bulletin de l' Acad. de Paris.*

### SOME RARE INCIDENTS IN CHRONIC BLENORRHŒA OF THE MALE URETHRA.

The pathological anatomy of chronic blenorrhœa in the male urethra has been hitherto very deficient, and therefore the thera peutical means are also defective. This is very easily explained by the very rare occurrence of death in persons affected with chronic blenorrhœa. The two following cases in which autopsy was made, throw some light on the pathological conditions of this disease. In both cases, there were excrescences on the surface of the urethra. These excresences have been very seldom referred to, but Morgagni had already mentioned them in A. D. 1745; also Hunter, 1835. Rokitanski mentions them especially as growths, substituting the callosities of the urethra, which have sometimes a papillar structure, and an appearance like condylomas. Birsh-Hirshfeld says that polypous excrescences are very rare in the male urethra, but more often found in that of females. The question whether these excrescences are true gonorrhœal warts, or whether they result from a so-called gonorrhœal abscess, is not yet solved, but the treatment would be accordingly a very different one. Our first case was that of a laborer forty-nine years of age, who began to suffer from blenorrhœa urethræ eighteen months before his death, and who had also balano-posthitis and peri-urethritis, followed by formation of abscess, which was opened through the perineum. A bougie of two mm. passed the urethra. At first the flow of urine and pus was free through the opening of the abscess, but soon infiltration of all the soft parts from the angulus penoscrotatus down to the tuber ischii followed, with gangrene of these parts. from the effect of which the patient died. The autopsy showed a stricture of the urethra in the highest degree, hypertrophy of the bladder, suppurative cystitis, and gangrenous peri-urethral infiltration. The urethra showed in the fossa scaphoidæ two elevated red spots two cm. in diameter, and contrasting by their color with the surface of the scaphoid fossa and the post-scaphoidal region. The region about the caput gallinaginis was discolored. The surface was rough, the most part in suppuration, and near the orificium urethræ there were excrescences. The microscopical examination showed excrescences in the scaphoid fossa, distinctly different from the normal papillæ. They consisted of hyaline granular substance, with some epithelium. On the surface of the postscaphoid part there were about seventeen excrescences, about 1 mm. high, of a fibrinous connective tissue, with cylindrical epithelium. The pars membranacea was filled with filiform excrescences, and here the mucous membrane looked like that of intestinal rugæ. They consisted of young connective tissue, which originated from the bundles of connective tissue running obliquely to the surface, so that the urethral walls looked like unravelled thread at some points. The warty excrescences showed in their interior part dense fibrinous connective tissue, lying sometimes in two different strata. The grouped excrescences and the brush-like, had different epithelium. The former were covered only by cylindrical epithelium, forming sometimes short angles. In the latter, the interstices were filled with flattened epithelium, which ran perpendicularly from the surface of the mucosa down to the roots of the excrescences. With these changes on the surface, there were analogous structures in the deeper layers. They consisted mostly in immigrations of round cells and new growths of connective tissue.—*Wiener Med. Wochenschrift.*

### PROLAPSUS OF THE LARGE INTESTINES 90CM. LONG THROUGH THE ANUS.

Miss J., twenty-five years of age, has had rachitis and infantile paralysis. There was anchylosis of the knee-joints and the upper parts of the limbs were paralyzed, so that the patient could walk only with crutches. There was constipation of the bowels for the last three years, which was treated by injections and salts. On the 26th of October, last year, she took an injection, which was followed by a semi-liquid evacuation. But when rising from the water-closet the patient felt a voluminous mass protruding from the anus. She thought it a hæmorrhoidal tumor, but the mother, looking at it, became alarmed, and sent for a physician. The patient did not complain of any pains, and asked the physician quietly to remove that nasty thing. The pulse was regular, and nothing indicated the terrible accident. In front of the anus was situated a massive tumor, globular on its superior part, cylindrical on its inferior and formed of transversal folds, characteristic of the large intestines. There was an orifice on the lowest part of the tumor, and the intestinal tunica had apparently taken part in the prolapsus, with invagination of the mucous membrane. The tumor was highly injected, and it was not possible to reduce it by taxis. For want of consultation, the operation was postponed until the next day. Vomiting occurred in the night, and colicky pains troubled the patient severely. In the morning the pulse was 130. The operation was performed by incision into the globular part of the tumor with an application of four silver sutures and by incision on the opposite side and application of three silk ligatures. The excised piece measured 80cm.; it consisted of the rectum and the S iliacum of the colon. The invagination was incomplete, and the tumor was like two tubes, one put into the other. The mucous membrane of the outer tube was brown and excoriated, that of the inner perfectly healthy. The tumor had undergone gangrene on its outer part. The patient died.—*Progrès Médical.*

### THE EFFECTS OF EXCITATION ON THE LINGUAL NERVE.

Faradic excitation of peripheric segments of the lingual nerve produces considerable dilatation of all the vessels on the center of the tongue; the latter take on a pronounced redness, the temperature rising at the same time. These phenomena appear even after ligature of the lingual artery and both carotids, and after dissection of the sympathetic nerve and excision of the upper cervical ganglion of the same side. When circulation is stopped by farado-puncture of the ventricles of the heart, the middle part and the opposite side of the tongue become pale and the vessels diminish, but these phenomena persist after the pulsation of the heart is stopped for a certain length of time. At the same time the vessels of the innervated side are dilated, those of the opposite side diminish. This latter fact is due to a vaso-constrictive action of the nerves. If it was due to a simple derivation, the blood-vessels would diminish, but the blood would not change its color, but this discolorization is stated in the various experiments. If, for instance, the segment of the right lingual nerve is faradized, the blood in the veins of the left side of the tongue becomes nearly black. This phenomenon remains for the time of ten minutes, while the faradized side will be reddened but for thirty seconds, which proves that the two processes are independent, one from the other.—*France Médicale.*

### CONGENITAL DOUBLE LUXATION OF THE FEMUR.

A primipara, thirty-five years of age, showed the following characteristic deformations: The great trochanters were situated more than 2cm. above the line of Nélaton, the femoral head was felt in the external iliac fossa, there was anteversion of the pelvis, and lumbar lordosis, no rachitis. The delivery of the child was accomplished easily by the forceps, but three days afterwards the woman died from septicæmia without inflammatory processes in the pelvic organs. At the femoral articulation, there were all the lesions due to congenital luxation. The cotyloid cavity was diminished to about three-fourths of its regular capacity, and it was generally atropied. The false articulation was formed in the upper and anterior part of the external iliac fossa, its circumference being about 6cm.; there was cartilage on the borders, and the Haversian canals were dilated. This lesion was on both sides. The neck of the femur was very small and of a conical form. The pelvis was asymmetrical. The promontorium was not in the median line, and there was atrophy of the left ilium. The lumbar column was lordosed. The most prominent deviation was in the transverse dimension, the antero-posterior diameters being also enlarged.—*L' Union Médicale.*

### HYDROPS SINUS FRONTALIS.

Mrs. D. A., 72 years of age, had a catarrh of the nose twelve years ago, which was followed by obstruction in the nares. The left eye was inflamed and successively dislocated downwards, the upper lid being immovable. In the upper angle of the orbit, there is a fluctuating tumor, in the deeper part of which is palpable the margin of bone. After incision, a large quantity of a serous pus is remeved. In the deeper part, a bone defect is visible, and this having been enlarged the finger can penetrate into the frontal cavity. A tube is inserted, and injections made into the cavity. In about two months the wound was healed. Erysipelas in the region of the scar followed after a month, and after incision large quantities of pus again poured out. A curved trocar was pushed from the frontal cavity into the left nasal cavity, and a tube inserted, one end of which projected from the wound, the other from the left nasal cavity. After several months, the tube was withdrawn, and a silk thread inserted. Recovery was then complete after a short time.—*Med. Chir. Centrblatt.*

FIVE CASES OF TRANSFUSION OF BLOOD INTO THE PERITONEAL CAVITY.

Ponfick has demonstrated by experiments that any quantity of defibrinated blood injected into the peritoneal cavity, will be absorbed with great benefit by any animal. Korzarowski, of Posen, has made these injections in five cases on the human person, with the best results. (1). Nephritis, articular affections, fever, profound anæmia. Two injections of 500 grammes of defibrinated blood into the peritoneal cavity, cure. (2). Nervosity, hysteria, spinal irritation and anæmia. One injection, radical cure. (3.) Phthisis well developed, after the first injection the appetite returns, fever and night-sweats disappear. (4). Anæmia, extreme weakness, patient in bed for three months. Eight days after injection of 600 grm., patient walks around; complete cure after three months. (5). Alcoholism, typhus exanthematicus, decubitus, pulmonary affection, 400 grm., injected, cure.—*L' Union Médicale.*

A NEW METHOD OF EMBALMING.

Instead of taking a solution of chloride of zinc, the following mixture is recommended: Thymol, 5 parts; alcohol, 45 parts; glycerine, 2,160 parts, and water, 1,080 parts. The injections made with this solution have the advantage of not being offensive, the instruments are not destroyed by it, and the bodies will be conserved any length of time, being mummified without putrefaction.—*Lyon Médicale.*

SARCOMA OF THE TESTICLE.

The patient, a strong man of forty years, has had a swelling of the right testicle for the last eight years. He was tapped first by a physician, after which operation the testicle grew smaller. Three years ago, he had a contusion of the testicle, and it swelled rapidly to the former size. He was then tapped twice without result. The tumor was as large as two fists, not painful and fluctuating in some places. The cord was normal, as was also the prostate. There has been no syphilis, and hydrocele was excluded. The operation was made in the usual way, three secondary hæmorrhages occurring. The microscopical examination proved the diagnosis to be sarcoma.—*Revue Médicale.*

### One Kidney.

In the autopsy of a young boy, nine years of age, there was found but one kidney (the left). The right kidney and also the right ureter was absent, as also the orifice into the bladder of this side. The left kidney was normal, its weight being 160 grammes. The arteries of this kidney were two in number, having a division on the left lateral part of the aorta, the upper one more voluminous than the inferior. In the neighborhood of this division, two small arteries were developed, which ran into the neighboring cellular tissue. The prostate was symmetrical, but there was but one seminal vesicle.—*Progrès Médical.*

### Stretching of the Median Nerve.

In hunting, a man was shot in the left arm. Paralysis of the forearm set in, with excessive pains. The piece of lead could not be found. The median nerve was paralyzed, and the muscles of the forearm and hand atrophied, the pains being intolerable. An incision 3 cm. long was made, the enlarged and indurated nerve laid bare and stretched. The wound healed, *prima intentione.* The pains were then moderated and more of a neuralgic character, and after a while they disappeared entirely. The paresis of the muscles diminished, and by galvanic treatment the patient was cured.—*Le Praticien.*

### Pilocarpine in Poliuria.

A patient suffering from poliuria azoturica used belladonna, brom. of potass., laudanum, injections of morphine and electricity without effect. Hypodermic injections of nitr. of pilocarpine, 0.20 in water 20.0, diminished the daily secreted urine from ten liters to two liters, and the quantity of urea was reduced from nine grm. to three grm. The weight of the body increased to eight kilogrm. in two months' time. In poliura glycosurica, the sugar disappeared after a short time. Fifteen injections will generally cure these diseases.—*H. Morgagni Giornale.*

THE following deaths from carbolic acid are reported: A man who had his hair clipped off was painted with carbolic acid over two-thirds of the head for some disease. He complained immediately about pains and dizzines in the head, became unconscious after a few minutes, and died shortly afterward. Three girls were painted for scabies all over the body with impure carbolic acid, and they became unconscious after a few minutes. Two carpenters had used carbolic acid against scabies; one of them cried suddenly, became intoxicated, and died after a few minutes; the other became unconscious, but he recovered. A child of fourteen months fell upon a bottle filled with a strong solution of carbolic acid; the bottle broke, and the child was covered with the acid. Death followed a few minutes afterward. A midwife applied a small compress soaked with carbolic acid to a suppurating spot on a little child, which died after two hours. Experiments on animals have proved that carbolic acid brought in contact with the bowels or peritoneum will produce shock and collapse.

BY a decree of the Russian minister of war, the Jewish military physicians in that country are banished from all central and divisionary administration, as also from all the large ports. They are not allowed to be stationed at Odessa, Kieff, Warsaw and Karkoff. All these Jewish physicians stationed there are discharged or sent to distant ports in Asia. A good many of these physicians have done excellent work in the last Turkish war. Oh Russia!

THE Congress of Hygiene and Demography was held at Geneva, from the 4th to the 9th of September. The great event was an elocutionary debate between Pasteur, of Paris, and Koch, of Berlin, but the latter not being able to master the French language, M. Pasteur remained the victor.

THE National Hygienic Congress of the Argentine Republic will be held at Buenos Ayres this month, as the *Revista Medico-Quirurgica* reports. The ..ost important question will be obligatory vaccination. During the last twenty-six years 6,755 persons have died from small-pox in Buenos Ayres, and in the country 11,635. A peremptory law will be introduced making vaccination obligatory.

Dr. OTTO SEIFERT, of Wuerzburg, reports that he had very good results in diphtheria from a solution of chinoline, 1.00; alcohol 50.0 and water 500.0; peppermint oil, two drops. The solution is used as a gargle, and the diphtheritic spots are painted with it. In all inflammations of the fauces the pains are stopped immediately by this solution.

THE Philosophical Society has opened its winter course of lectures, and a paper likely to interest medical men will be delivered January 20, at 8 P. M., by Dr. J. S. Jewell; subject, "Heredity, Mental and Physical." The society meets every Saturday evening at Apollo Hall, State street, corner Randolph.

THE Medical Society of Berlin energetically protests against the new sanitary institution called forth by Esmarch, of Kiel, and the Berlin physicians understand a good deal about this matter, as American surgeons understand somewhat of surgery.

*L'Union Médicale*, in reporting the first resection of the stomach in Italy, executed by Azzio Caselli, and which was folowed by immediate death, calls the operation an "inutile traumatisme."

A HOMŒOPATHIC physician in Switzerland advertises that he performs "surgical operations *milder* than any physician of the other school." At last we know what a homœopathic surgeon is.

THE leaves of euphorbia pilulifera L., a plant growing in Queensland, are reported to be a sure remedy for asthma and other affections of the chest.

